# Carotid Stenting as Definitive Treatment for Free Floating Thrombus—Review of 7 Cases

**DOI:** 10.1007/s00062-020-00898-y

**Published:** 2020-03-27

**Authors:** P. Bhogal, M. AlMatter, M. Aguilar Pérez, H. Bäzner, H. Henkes, V. Hellstern

**Affiliations:** 1 Department of Interventional Neuroradiology, The Royal London Hospital, Whitechapel Road, E1 1BB London, UK; 2 Neuroradiologische Klinik, Neurozentrum, Klinikum Stuttgart, Stuttgart, Germany; 3 Neurologische Klinik, Neurozentrum, Klinikum Stuttgart, Stuttgart, Germany; 4 Medical Faculty, University Duisburg-Essen, Essen, Germany

**Keywords:** Free floating thrombus, Atherosclerosis, Carotid stenting, Carotid

## Abstract

**Background and Purpose:**

Free floating thrombus (FFT) is a rare condition. The optimal treatment strategy is yet to be determined although medical management with anticoagulation is the mainstay. This article reports experience of treating FFT with carotid stenting.

**Methods:**

A retrospective analysis of a prospectively maintained database was performed to identify all patients with FFT treated with carotid stenting. For each patient the demographic data, clinical presentation, location of the thrombus, type of stent and use of adjunctive devices, e.g. balloon guide catheters, clinical and radiological follow-up information as well as complications were recorded.

**Results:**

A total of 7 patients, 4 female, with mean age of 55.6 ± 14.5 years were identified. The median National Institutes of Health Stroke Scale (NIHSS) was 7 (range 0–13) at presentation. Free floating thrombus was seen on the left in the majority of cases (*n* = 6, 85.7%). None of the patients had intracranial large vessel occlusion. The FFT was located in the CCA in 2 cases (28.6%) and the proximal ICA in the remaining 5 cases (71.4%). The Wallstent was used in 5 patients and a cGuard stent used in 2 patients. In 1 patient 2 overlapping stents were used but a single stent was used in the remaining patients. In 6 cases a distal filter wire was used and in 2 cases a balloon guide catheter was used as embolic protection. There were no intraoperative complications and no cases of distal clot migration or intracranial large vessel occlusion during the procedure. At last follow-up (*n* = 7) 6 patients were recorded as modified Rankin Scale (mRS) ≤2 and 1 patient was mRS 3.

**Conclusion:**

Free floating thrombus of the carotid arteries can be managed with stenting.

## Introduction

Free floating thrombus (FFT) in the carotid artery is defined as an elongated thrombus attached to the arterial wall with circumferential blood flow around its most distal aspect, with cyclical motion relating to the cardiac cycle [1]. It is a rare condition that typically presents acutely but can also present sub-acutely [2]. The internal carotid artery is the most commonly affected vessel and atherosclerosis is recognized as the most commonly associated pathology [3].

The optimal treatment strategy has not been defined, at least in part due to the rarity of the condition, and comparative studies between medical management (anticoagulation and/or antiplatelet) and surgical treatment (carotid stenting, endarterectomy or bypass procedure) are lacking.

This article presents a series of 7 cases all treated with stenting and trapping of the thrombus between the stent and the arterial wall.

### Methods

#### Patient Population

We performed a retrospective review of our prospectively maintained database to identify all patients with FFT in the vessels of the anterior circulation and treated with carotid stenting. We searched our database from January 2008 to September 2019.

For each patient we recorded demographic data, clinical presentation, location of the thrombus, type of stent and use of adjunctive devices, e.g. balloon guide catheters, clinical and radiological follow-up information as well as complications. Patient informed consent was obtained in written form, before the procedure in cases where the patients was deemed suitable to provide consent. In emergency cases consent was based on best medical opinion and whenever possible discussion with family and next of kin.

#### Endovascular Treatment

All treatments were performed with the patient under general anesthesia. All patients received dual anti-platelet treatment (DAPT), aspirin 500 mg IV and ticagrelor 180 mg or clopidogrel 600 mg p. o., on the morning of the procedure. The effectiveness of the antiplatelet regimen was tested using the VerifyNow (Accumetrics, Bedford, MA, USA) approximately 4–6 h afterwards. Postprocedurally all patients were placed on DAPT for at least 6 months.

All procedures were performed via the right common femoral artery using an 8F access system. Either proximal protection via a balloon guide catheter or distal embolic protection via a filter wire was used in all cases. Balloon angioplasty of the thrombus prior to stent implantation was not performed as it was believed this may fragment the thrombus and result in intracranial occlusion. All procedures were performed with the patient under heparin anticoagulation with a 5000 IU bolus dose at the start of the procedure and subsequent 1000 IU bolus doses (if required) every hour to maintain the activated clotting time (ACT) between 2–2.5 times the baseline level.

Either a Carotid Wallstent (Boston Scientific, Marlborough, MA, USA) or cGuard carotid stent (InspireMD, Boston, MA, USA) were used. The choice between the stents was based on operator preference and availability.

#### Procedural Assessment and Follow-Up

Patency and flow characteristics within the parent vessel and within the distal intracranial circulation was assessed angiographically immediately after placement of the carotid stent. Magnetic resonance imaging (MRI) was performed at 24 h in those patients that could undergo MRI. In those patients that could not undergo MRI a computed tomography (CT) and CT angiography (CTA) were performed to ensure adequate patency of the stents and no new ischemia.

Neurological examinations were performed to evaluate for potential ischemic or hemorrhagic complications in the postoperative period (<24 h postprocedure) and at each subsequent follow-up. The modified Rankin Scale (mRS) was documented at discharge and at 90 days for patients that could attend follow-up appointments.

## Results

During the time period searched we performed approximately 2200 carotid stenting procedures. We identified 7 patients, 4 female, with mean age of 55.6 ± 14.5 years. The patients presented with a variety of symptoms including both persistent and transient neurological symptoms. The median National Institutes of Health Stroke Scale (NIHSS) was 7 (range 0–13) at presentation, four patients underwent MRI and three patients underwent CT imaging of the brain and cervical vessels. Microembolic ischemic events were seen in 5 patients, a territorial stroke was seen in 1 patient and no acute changes were seen in the remaining patient. Intracranial large vessel occlusion was not seen in any of the patients. An FFT was seen in all patients with the FFT seen on the left in the majority of cases (*n* = 6, 85.7%). The FFT was located in the CCA in 2 cases (28.6%) and the proximal ICA in the remaining 5 cases (71.4%). The time of symptom onset was unknown in 3 patients and 2 patients woke up with symptoms. The median time from symptom onset to treatment in the remaining two patients was 388.5 min (range 371–406 min). The results are summarized in Table 1.

**Table 1:** Baseline characteristics, clinical details, procedural details and outcome

Patient No	Demographic		Admission Status				Imaging modality and Findings			Treatment						Anti-platelet and Anti-coagulation regime			Follow-Up	
	Age	Sex	Symptoms	Admission mRS	Onset	NIHSS on admission	Imaging Modality	Imaging findings	Thrombus Location	Time of Treatment	Symptom onset to treatment time (mins)	Stent	Filter	Balloon guide catheter	Complications	Medication for EVT	Post-op Daily medication	mRS at discharge	Radiological new ischaemic change	mRS at 90 days
1	59	f	TIA	1	1300hrs	0	CTA	Embolic microischaemia	Left proximal ICA	19:11	371	1 × Wallstent	No	Yes (Flowgate)	None	500 mg ASA, 180 mg Ticagrelor, bolus of epitifibatide	75 mg ASA OD, 90 mg Ticagrelor BD	0	NA	0
2	58	f	Paresis of the right arm, facial paresis, neglect	4	1100hrs	13	CTA	Territorial stroke—no intracranial LVO	Left proximal ICA	17:46	404	1 × Wallstent	Yes (SpiderX 6F)	No	None	500 mg ASA, 180 mg Ticagrelor, bolus of epitifibatide	75 mg ASA OD, 90 mg Ticagrelor BD	2	NA	2
3	64	m	Transient right sided hemiparesis	2	Wake-up	1	MRA	Embolic microischaemia	Left CCA	12:25	Wake-up	2 × Wallstent	Yes (SpiderX 6F)	No	None	500 mg ASA, 180 mg Ticagrelor, bolus of epitifibatide	75 mg ASA OD, 90 mg Ticagrelor BD	2	No	2
4	33	f	Hypoasthesia of the right hand	2	Unknown	0	CTA	No acute intracranial findings	Left proximal ICA	18:22	Unknown	1 × cGuard	Yes (SpiderX 6F)	No	None	500 mg ASA, 180 mg Ticagrelor	75 mg ASA OD, 90 mg Ticagrelor BD	2	NA	0
5	66	m	Transient right sided hemiparesis	3	Unknown	8	MRA	Embolic microischaemia	Left proximal ICA	11:24	Unknown	1 × Wallstent	Yes (SpiderX 7F)	No	None	500 mg ASA, 180 mg Ticagrelor, bolus of epitifibatide	75 mg ASA OD, 90 mg Ticagrelor BD	2	No	2
6	38	f	Paresis of the right arm, aphasia, visual symptoms	4	Wake-up	12	MRA	Embolic microischaemia	Left CCA	15:13	Wake-up	1 × cGuard	Yes (SpiderX 6F)	No	None	500 mg ASA, 180 mg Ticagrelor, bolus of epitifibatide	75 mg ASA OD, 90 mg Ticagrelor BD	5	Yes	2
7	71	m	Dysarthria, paresis of the arm, facial paresis	4	Unknown	7	MRA	Embolic microischaemia	Right proximal ICA	09:20	Unknown	1 × Wallstent	Yes (SpiderX 7F)	Yes (Cello)	In-Stent- thrombosis- requiring repeat treatments	500 mg ASA, 600 mg Clopidogrel	75 mg ASA OD, 75 mg Clopidogrel OD	4	No	3

*ASA* acetylsalicylic acid (aspirin), *BD* bidaily, *CCA* common carotid artery, *ICA* internal carotid artery, *LVO* large vessel occlusion, *OD* once daily, *TIA* transient ischaemic attack

All patients were treated with carotid stenting and the Wallstent was used in 5 patients with the cGuard stent being used in 2 patients. In 2 patients 2 overlapping stents were used but a single stent was used in the remaining patients. In six cases a distal filter wire was used and in two cases a balloon guide catheter was used as embolic protection. There were no intra-operative complications and no cases of distal clot migration or intracranial large vessel occlusion during the procedure. Clot protrusion could be seen in one case and this necessitated a second stent deployment, which resulted in good coverage of the FFT. In one case in-stent thrombosis occurred and this necessitated a repeat procedure (see later).

There was post-operative imaging available in 4 patients with no evidence of new ischaemic change seen in 3 (75%). New microembolic infarction was seen in 1 patient. At discharge 5 patients had a good outcome (mRS ≤2), one patient was discharged at mRS 4 and one at mRS 5. At last 90 days follow-up (*n* = 7) 6 patients were recorded as mRS ≤2 and one patient was mRS 3. A single patient was discharged with a worse mRS than at admission (patient 6) however, at 3 months they had made a significant clinical improvement (mRS 2).

### Case Example

**Fig. 1:**
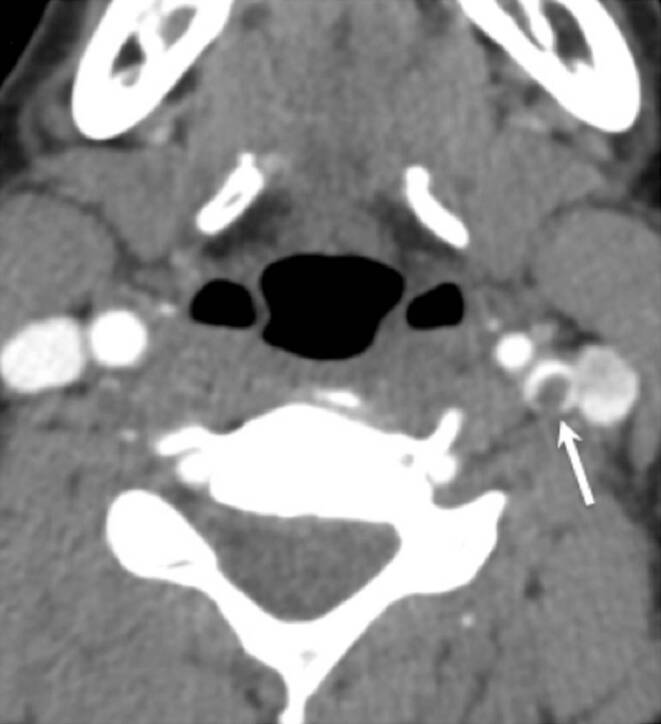
Cross sectional CT angiogram of the neck showing free floating thrombus in the proximal ICA resulting in severe stenosis of the ICA (*white arrow*)

A 33-year-old female patient with a background history of HIV presented with symptoms of acute hypesthesia of the right hand and lip, which was suspected to be caused by either stroke or possibly acute demyelination. A head CT and CTA were performed showing no acute ischemia but a high-grade stenosis of the left ICA due to a FFT (Fig. 1).

**Fig. 2:**
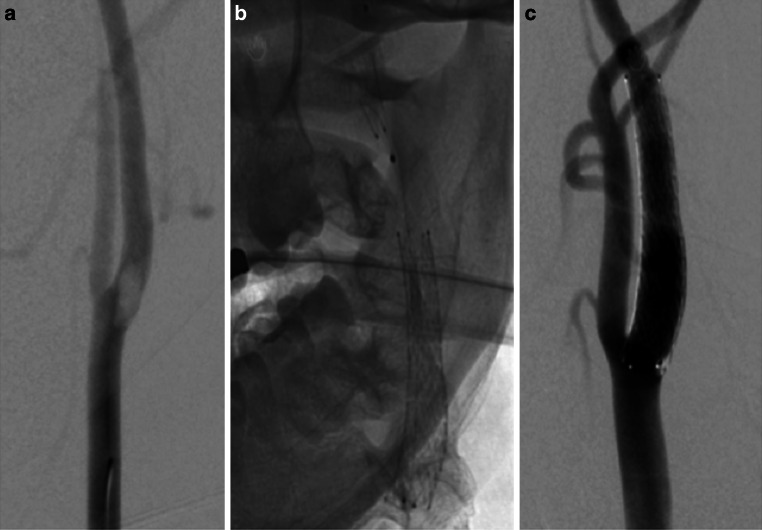
Catheter angiography revealed a large FFT in the proximal ICA that resulted in some flow restriction distally (**a**). There was no evidence of intracranial occlusion (not shown). A carotid stent (cGuard, 7 × 40 mm) was placed over the FFT with embolic protection using a distal filter wire (**b**). Postprocedural angiography showed entrapment of the FFT and restoration of the ICA lumen with good distal flow (**c**)

After general anesthesia catheter angiography confirmed an FFT in the left ICA. Using a standard 8 Fr access and distal protection device a single cGuard carotid stent was implanted without complication (Fig. 2).

**Fig. 3:**
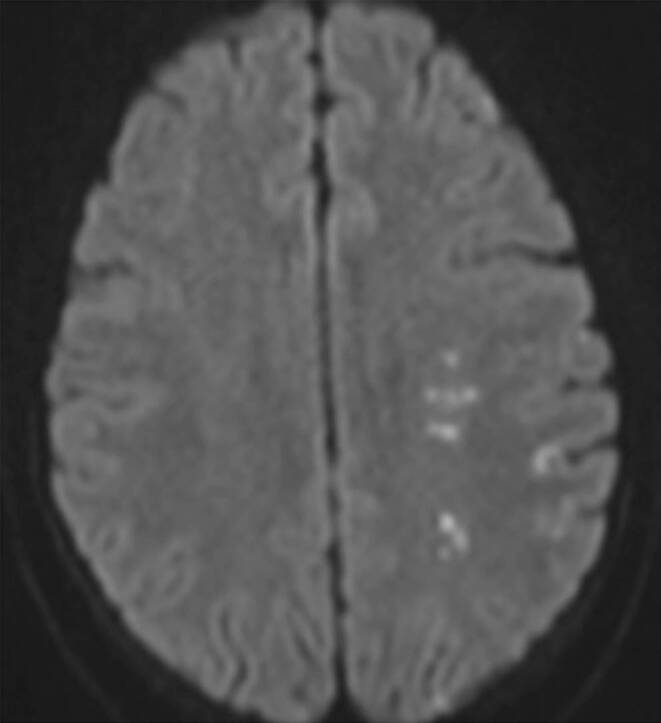
Several small microembolic or possible watershed infarcts were seen on diffusion-weighted imaging. It was unclear if these had occurred prior to the procedure or post-procedure as an MRI scan had not been performed preoperatively. The patient was asymptomatic post-procedure and at last assessment remained at mRS 0

Postprocedural MRI and prior to discharge showed restricted diffusion thought to be secondary to microemboli (Fig. 3). It was unclear if these were due to the treatment itself or were present prior to the procedure. The patient was asymptomatic at discharge and at last follow-up remained mRS 0.

### In-Stent Thrombosis Case

In our series a single patient required repeat treatment. The patient presented with a worsening left-sided hemiparesis, facial paresis, and dysarthria with an NIHSS of 14. On CT angiography a clot was seen in the cervical ICA that resulted in approximately 75% stenosis but there was no evidence of intracranial large vessel occlusion. Initially the patient refused endovascular treatment and was treated with dual anti-platelet therapy. The patient’s symptoms improved and the NIHSS decreased to 7; however, angiography performed 9 days later showed progression of the clot which was now 90% occlusive and after discussion with the patient endovascular treatment was performed and a Carotid Wallstent was implanted. The treatment was uneventful and the patient was discharged 3 days later. An ultrasound performed 3 months later showed thrombus formation within the stent, which was confirmed on catheter angiography. This was causing 50% stenosis of the vessel and there was evidence of stent strut fracture at the site of the thrombus. Anti-platelet testing confirmed suitable levels of anti-platelet activity. A second Wallstent was telescoped inside the first stent with the hope of trapping the thrombus once again. A further delayed angiogram performed 2 months demonstrated repeat thrombus formation and on this occasion a cGuard stent was implanted following which there was no further evidence of thrombus formation.

## Discussion

Carotid FFT is an uncommon condition and among stroke patients it has and estimated frequency of 0.4% [4]. Although atherosclerosis is recognized as one of the most common causes of FFT, an association with other conditions has also been documented including fibromuscular dysplasia, trauma, external compression, dissection and vasculitis [1, 5–8]. Carotid FFT is associated with a high short-term risk of recurrent embolic ischemic events [2, 9] and hence careful consideration of the optimal treatment strategy, which may vary between patients, is required. Various different treatment strategies including pharmacotherapy, endovascular, and open surgical, have been described in the literature in addition to combined techniques.

From a medical perspective the principle treatment options consist of anti-coagulation, anti-platelet therapy or thrombolysis. In the recent systematic literature review of Fridman et al. [10]. The primary endpoint in this analysis was a composite of any stroke, transient ischemic attack (TIA), silent brain ischemia on diffusion-weighted MRI scan, or death at 30 days after the index event. The authors showed that patients receiving intravenous tissue plasminogen activator (IV-tPA) had an increased risk of recurrent ischemic stroke in the first 24 h compared to those who did not receive IV-tPA (hazard risk, HR 14.79; 95% CI 3.41–64.25, *p* < 0.0001). This presumably occurs secondary to clot fragmentation and dislodgement similar to that which has been reported after thrombolysis in myocardial infarction or ischemic stroke patients with calcified thrombus [11]. In the literature review performed earlier by Bhatti et al. [1] complete dissolution of the FFT, without any further neurological deterioration, was seen in 86% of patients. In this study patients were initially managed with heparin with subsequent conversion to warfarin for several weeks to months. Even though medical management appears to be the mainstay of treatment there is a lack of consensus regarding the optimal medical regimen and whether this should include antiplatelet agents, anticoagulants, or both. Similarly, there is no consensus on how long treatment should be continued for with at least one report documenting the recurrence of thrombus after discontinuation of anticoagulation treatment [12]. This is further compounded by the variation in response to different antiplatelet agents.

Surgical treatment offers the potential advantage of a definitive treatment for the FFT via embolectomy and also the underlying pathology via endarterectomy in cases of atherosclerosis. Endovascular treatment options have more recently been shown to be effective in the management of FFT [13–15]. Potential advantages of the endovascular approach compared to the standard vascular surgical approach include shorter procedure times with minimal vascular manipulation, which has the potential for dislodging the FFT, but still having the potential to cover the FFT en bloc. General anesthesia can also be avoided and advanced techniques honed from other procedures, for example flow arrest with balloon guide catheters and the use of distal filter wires, can be adopted to minimize the risk of distal embolization intraoperatively. Similarly, if distal embolization does occur during the procedure then an intracranial mechanical thrombectomy can be performed immediately. In our opinion early revascularization should be considered for cases where there is evidence of flow restriction as well as recurrent symptoms despite medical management.

Chakhtoura et al. [16] were the first to report the use of carotid stenting for the treatment of FFT. In these two cases a carotid Wallstent was implanted with patency of the stents on follow-up imaging and no recurrence of symptoms. In 2005 Parodi et al. [17] described a case of FFT treated with carotid stenting using the Wallstent where medicinal management with anti-coagulation initially failed. In this case the authors initially tried to aspirate the FFT in conjunction with the Parodi anti emboli system (PAES) (ArteriA, San Francisco, CA, USA); however, this failed and a stent was placed that resulted in trapping of the FFT. On follow-up imaging, with carotid duplex ultrasound, the proximal end of the stent was not completely opposed to the wall of the CCA and there was concern the FFT was not adequately constrained. In order to ensure the FFT was completely trapped the authors deployed a second carotid stent to cover the proximal end of the first stent.

Mechanical thrombectomy, using either aspiration or stent-retrievers, can also be performed and several cases have been published [13, 15, 18]. In the small series (*n* = 3) presented by Fitzpatrick et al. [15] the authors used a combined approach with proximal balloon protection, distal protection with a filter wire, and stent-retriever mechanical thrombectomy. Using this technique the authors successfully retrieved all the clots, with no migration into the intracranial circulation with a maximum of two passes. Giragani et al. [13] described a similar approach, combining a distal filter wire and stent-retriever. In this case, despite medical treatment with antiplatelets and anticoagulants, the patient continued to have TIA and therefore, a more definite option was considered. Uniquely, the authors describe the concomitant use of ultrasound to confirm adherence of the clot to the stent-retriever intraoperatively and they describe complete removal of the clot after 2 passes.

In our own series we used both the Wallstent and the cGuard and although both devices appear to be suitable for this procedure we believe that stents with a micromesh may offer a better safety profile and prevent clot fragmentation and distal migration; however, this requires further study. Complete coverage of the FFT is essential. Given the elastic nature of FFT a change in the length of the FFT after stent deployment could be encountered. For these reasons we believe that longer stents should be used to ensure complete coverage of the FFT both proximally and distally even if there is a conformational change in the FFT.

Its retrospective design and small numbers limit our study. Not all patients underwent preoperative and postoperative MRI and therefore small new ischemic events may have gone undetected. Similarly, the lack of a control group for comparison with medical management limits the generalizability of the technique.

## Conclusion

For patients with a symptomatic free floating thrombus of the carotid arteries stenting can offer a definitive treatment option with good safety profile.
